# F235. DIFFERENTIAL EFFECTS OF ANTIPSYCHOTICS ON NEUROINFLAMMATION AND ENERGY SENSING IN A HYPOTHALAMIC CELL LINE

**DOI:** 10.1093/schbul/sby017.766

**Published:** 2018-04-01

**Authors:** Chantel Kowalchuk, Pruntha Kanagasundaram, Gary Remington, Denise Belsham, Margaret Hahn

**Affiliations:** 1 Centre for Addiction and Mental Health, University of Toronto; 2 University of Toronto

## Abstract

**Background:**

Antipsychotics (AP)s are the cornerstone of treatment for schizophrenia but cause serious metabolic side-effects. The hypothalamus is the primary brain region responsible for whole body energy regulation and disruptions in energy sensing (e.g. insulin signaling) and inflammation in this brain region have been implicated in the development of peripheral insulin resistance and obesity. Thus, it is possible that hypothalamic inflammation and disturbed energy sensing could be involved in AP-induced metabolic disturbances. Data in relation to AP-associated changes in inflammatory markers in schizophrenia has been inconsistent, owing in part to confounds of illness-related factors (e.g. diet, smoking) and secondary effects of weight gain. To our knowledge, direct effects of APs on hypothalamic cells in relation to insulin signaling and inflammation have not been examined.

**Methods:**

To examine direct, molecular effects of APs in the hypothalamus, an immortalized rat hypothalamic cell line, rHypoE-19, was treated with olanzapine (dose range between 0.25–100 uM), clozapine (2.5–100 uM) or aripiprazole (5–20 uM). Western blotting was used to detect changes in the energy sensing protein AMPK, components of the insulin signaling pathway (AKT, GSK3B), and components of the mitogen activated-protein kinase (MAPK) pathway (ERK1/2, JNK, p38), the latter which are linked to inflammation. Quantitative real-time PCR was performed to determine changes in the mRNA expression of interleukin (IL)-6, IL-10 and brain derived neurotrophic factor (BDNF).

**Results:**

Both olanzapine (100 uM) and clozapine (100 uM) significantly increased pERK1/2 and pJNK protein expression, while aripiprazole (20 uM) only increased pJNK. Clozapine (100 uM) and aripiprazole (5 and 20 uM) significantly increased AMPK phosphorylation and inhibited insulin-induced phosphorylation of AKT. Olanzapine (100 uM) treatment caused a significant increase in IL-6 while aripiprazole (20 uM) significantly decreased IL-10. Olanzapine (100 uM) and aripiprazole (20 uM) increased BDNF expression.

**Discussion:**

All the APs studied upregulated pJNK, along with olanzapine-associated increases in IL-6, and aripiprazole-associated decreases in IL-10, together suggesting AP-mediated upregulation of pro-inflammatory pathways in rHypoE-19 neurons. Aripiprazole and clozapine (but not olanzapine) inhibited insulin-stimulated AKT, suggesting impaired hypothalamic insulin action by some, but not all, APs. Clozapine additionally increased AMPK phosphorylation (activation), an orexigenic energy sensor, which would also be expected to disrupt energy homeostasis. Conversely, olanzapine and aripiprazole increased BDNF, a factor linked to the underlying etiology of schizophrenia, suggesting BDNF upregulation may be a mechanism of therapeutic action. Taken together, our findings suggest differential and pleotropic effects of APs on neuroinflammation and energy sensing in the hypothalamus, which do not necessarily align consistently with known metabolic liability of these agents (i.e. clozapine = olanzapine > aripiprazole). Our data warrants further exploration into the mechanism of these effects, including replication of these effects in an in vivo model.

